# The transmission dynamics of Middle East Respiratory Syndrome coronavirus

**DOI:** 10.1016/j.tmaid.2021.102243

**Published:** 2022

**Authors:** Jia Rui, Qiupeng Wang, Jinlong Lv, Bin Zhao, Qingqing Hu, Heng Du, Wenfeng Gong, Zeyu Zhao, Jingwen Xu, Yuanzhao Zhu, Xingchun Liu, Yao Wang, Meng Yang, Li Luo, Qiuping Chen, Benhua Zhao, Yanhua Su, Jing-An Cui, Tianmu Chen

**Affiliations:** a State Key Laboratory of Molecular Vaccinology and Molecular Diagnostics, School of Public Health, Xiamen University, Xiamen City, 361102, Fujian Province, People's Republic of China; b Department of Mathematics, School of Science, Beijing University of Civil Engineering and Architecture, Beijing, 102616, People's Republic of China; c State Key Laboratory of Molecular Vaccinology and Molecular Diagnostics, Laboratory Department, Xiang'an Hospital of Xiamen University, Xiamen City, Fujian Province, People's Republic of China; d Division of Public Health, School of Medicine, University of Utah, 201 Presidents Circle, Salt Lake City, 84112, Utah, USA; e The Bill & Melinda Gates Foundation, Beijing, 100027, People's Republic of China; f The Bill & Melinda Gates Foundation, Seattle, 98103, WA, USA; g Medical Insurance Office, Xiang'an Hospital of Xiamen University, Xiamen City, Fujian Province, People's Republic of China; h School of Journalism and Communication, Peking University, Beijing, 100871, People's Republic of China

**Keywords:** Middle East respiratory syndrome, Coronavirus, Mathematical model, Basic reproduction number

## Abstract

**Background:**

In this study, we aimed to quantify the contribution of different transmission routes of the Middle East respiratory syndrome (MERS) and determine its transmissibility.

**Methods:**

Based on the natural history and transmission features of MERS in different countries, a susceptible-exposed-symptomatic-asymptomatic-recovered/death (SEIARD) model and a multi-route dynamic model (MMDM). The SEIARD model and MMDM were adopted to simulate MERS in South Korea and Saudi Arabia, respectively. Data on reported MERS cases in the two countries were obtained from the World Health Organization. Thereafter, the next generation matrix method was employed to derive the equation for the basic reproduction number (*R*_0_), and the model fitting procedure was adopted to calculate the *R*_0_ values corresponding to these different countries.

**Results:**

In South Korea, ‘Person-to-Person’ transmission was identified as the main mode of MERS transmission in healthcare settings, while in Saudi Arabia, in addition to ‘Person-to-Person’ transmission, ‘Host-to-Host’ and ‘Host-to-Person’ transmission also occurred under certain scenarios, with camels being the main host. Further, the fitting results showed that the SEIARD model and MMDM fitted the data well. The mean *R*_0_ value was 8.59 (95% confidence interval [CI]: 0–28.02) for MERS in South Korea, and for MERS in Saudi Arabia, it was 1.15 and 1.02 (95% CI: 0.86–1.44) for the ‘Person-to-Person’ and ‘Camel-to-Camel’ transmission routes, respectively.

**Conclusions:**

The SEIARD and MMDM model can be used to simulate the transmission of MERS in different countries. Additionally, in Saudi Arabia, the transmissibility of MERS was almost the same among hosts (camels) and humans.

## 1 Introduction

The Middle East respiratory syndrome coronavirus (MERS-CoV), which belongs to the family of coronaviruses like the novel coronavirus, was first detected in a hospital in Jordan in April 2012. It caused two large-scale MERS outbreaks in Saudi Arabia in 2013 and one in South Korea in 2015 [1]. Most patients with MERS develop severe respiratory illnesses with fever, cough, and shortness of breath [2], and unfortunately, no vaccine or specific treatment method is currently available in this regard [3].

According to the World Health Organization (WHO) [4], as of November 2019, 27 countries worldwide reported a total of 2494 laboratory-confirmed MERS cases, and approximately 35% of these patients with MERS-CoV infection died. The reporting countries are mainly distributed in the Middle East region, especially in the Arabian Peninsula; however, of recent, sporadic imported cases have recently been reported in Europe, North America, Africa, and Asia [5]. The largest outbreak outside the Middle East region was that which occurred in South Korea in 2015, which was declared to be over in 2018 [6]. Overall, 186 people were infected, 38 deaths were recorded (20.43% fatality rate), and 16,752 people had to adopt compulsory quarantine measures. In China, the first imported confirmed MERS case, someone who had been in close contact with a confirmed case in South Korea, was detected in Huizhou City, Guangdong Province.

Our search of existing literature revealed that most relevant studies on MERS have been focused on experimental research at biological level, whereas epidemiological studies, in which mathematical models were used to study the transmission dynamics of MERS, are scarce. For the 2015 epidemic in South Korea, we found that some researchers had constructed models, such as the Susceptible-Exposed-Infectious-Isolated-Removed (SEIJR) model [7], Susceptible-Exposed-Infectious-Asymptomatic-Hospitalized-Removed (SEIAHR) model [8], and Susceptible-Exposed-Infectious-Confirmed (SEIC) model [9], and for the 2012 epidemic in Saudi Arabia, we noted that the SEIAR model [10] and the SEIAHR model [11] had been constructed; however, most of these modelling studies were limited to person-to-person transmission. Recent studies have shown that MERS-CoV is not yet fully adapted to infect humans, implying that terminating person-to-person transmission is not an effective response to MERS [12]. However, no research or mathematical modelling has considered transmission routes such as camel-to-camel, camel-to-person, or person-to-person.

Therefore, in this study, we developed the susceptible-exposed-symptomatic-asymptomatic-recovered/death (SEIARD) model and the multi-route dynamic model (MMDM), both of which consider three transmission routes (person-to-person, host-to-host, and host-to-person). Thereafter, the models were used to fit the data collected from the WHO website and calculate the transmissibility of MERS based the abovementioned three transmission routes.

## 2 Materials and methods

### 2.1 Data collection and processing

In this study publicly available data [13], recorded by WHO, were used. Since the MERS outbreak in September 2012, WHO has received notifications regarding confirmed MERS-CoV cases from 27 countries. Further, until the data collection deadline (December 18, 2019) for this study, countries in the Middle East continued to report sporadic confirmed MERS-CoV cases monthly to WHO during the COVID-19 pandemic. By comparing this data with the publicly available information reported by the Ministries of the two countries (Saudi Arabia and South Korea), in this study, we included MERS cases reported in South Korea between 11 May and July 2, 2015 and those reported in Saudi Arabia between February 24, 2013 and 29 October 2019.

Patient data, including gender, age, region, comorbidities, occupation, e.g., health care worker (HCW), severity of disease, date of death, date of laboratory confirmation, date of symptom onset, and contact history, were also retrieved and used as input data for the models. Furthermore, according to the MERS epidemic report [13] published on December 18, 2019:

(1) Overall, 187 MERS cases with detailed data were recorded in South Korea. All these cases were new cases reported after January 5, 2015, when WHO began using standardised case reporting. We also noticed that two cases were reported on October 11, 2015 and August 28, 2018. However, both were imported cases and did not cause a large-scale epidemic. Therefore, they were excluded from the study.
(2) Overall, 1469 MERS cases with detailed data were recorded in Saudi Arabia. This included five patients with mild symptoms, 73 asymptomatic patients, 66 cases were reported as “‘Not Available (NA)’, 37 cases recorded as ‘No Report (NR)’, and three cases with blurred information. Therefore, 1285 cases, with a valid ‘date of symptom onset’, were included in the Saudi Arabia database.

As of December 5, 2019, WHO had recorded 2446 laboratory-confirmed MERS cases from 27 countries worldwide (Table .1). The top six countries, most of which are in the Middle East region, were Saudi Arabia (2,046), South Korea (187), United Arab Emirates (92), Jordan (27), Oman (24), and Qatar (20). Additionally, the remaining countries had scattered imported cases with no evidence of a potential MERS-CoV outbreak.

**Table 1:** MERS-outbreak reported countries and number of reported cases.

Reported countries	Number of reported cases	Reported countries	Number of reported cases	Reported countries	Number of reported cases
Austria	2	Iran	6	Philippines	2
Algeria	2	Italy	3	Qatar	20
Bahrain	1	Jordan	27	South Korea	187
China	1	Kuwait	4	Saudi Arabia	2046*
Egypt	1	Lebanon	2	Spain	1
France	2	Malaysia	2	Thailand	3
Germany	2*	Netherlands	2	Tunisia	3
Greece	1	Oman	24	Turkey	1
UAE	92	UK	5	U·S.	3
Yemen	1				
				Total	2446

Note: (1) One of the two cases in Germany was reported by The Robert Koch Institute, so it was included in Germany.

(2) Kingdom of Saudi Arabia contains 571 cases of missing data due to a general description that failed to capture case information.

### 2.2 Transmission routes of MERS-CoV

The transmission routes of MERS-CoV include ‘Host-to-Host’, ‘Host-to-Person’, and ‘Person-to-Person’. Even though studies have shown that person-to-person transmission of MERS-CoV accounts for approximately 60% of all cases [11], hospitals and families are regarded as centres of MERS-CoV outbreaks [5]. However, some studies have demonstrated that it is unlikely that the MERS outbreak in Saudi Arabia resulted from a continuous human-to-human transmission chain [14]. Camels are one of the principal hosts of MERS-CoV; thus, they may be the main source of human infections [15,16].

**Fig. 1:**
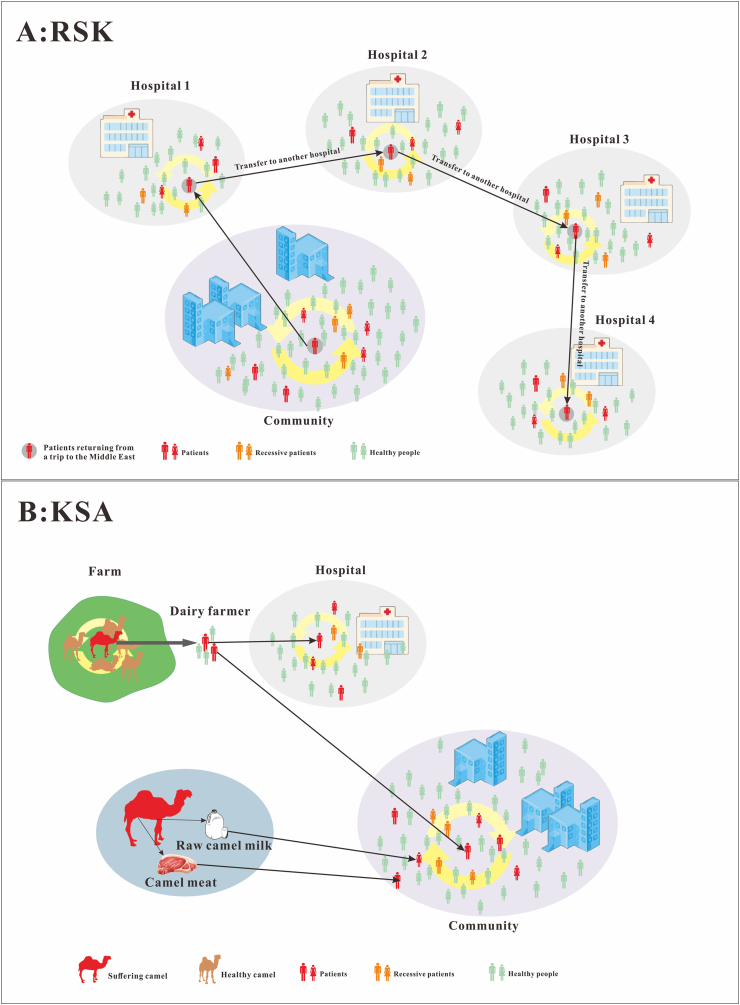
Schematic diagram of the MERS-CoV transmission route (A, South Korea; B, Saudi Arabia).

As shown in Fig. 1-A, the 2015 MERS outbreak in South Korea originated from one imported case (identified as the first case) travelling from the Middle East region that caused subsequent hospital and community (family) transmission. This indicates that the spread of MERS-CoV during the epidemic in South Korean was predominantly via “person-to-person” transmission.

Fig. 1-B demonstrates that in addition to the ‘person-to-person’ transmission observed during the epidemic in South Korea, the large camel population in the Middle East (Table 2) plays a significant role during the epidemics in Saudi Arabian (See detail in Supplemental file). Although the transmission from animals to humans is not fully understood, it has been demonstrated that MERS-CoV is a zoonotic virus, with dromedary camels as the chief natural host [15,17]. Moreover, other livestock species (including cattle, sheep, and goats) or other animals (including wildlife) could also be involved in the transmission of MERS-CoV [17]. Reportedly [15,18], MERS-CoV strains with the same sequence as human strains have been isolated from dromedary camels in several countries, including Egypt, Oman, Qatar, and Saudi Arabia, where people such as farm workers have frequent contact with infected camels positive for MERS-CoV antibodies [19]. Direct or indirect contact between humans and camels such as airborne spread [20], the consumption of MERS-CoV-contaminated camel meat [16], and the consumption of raw camel milk [21] can cause repeated MERS-CoV infections in the population [22].

**Table 2:** Human population and camel population in KSA from 2011 to 2019.

Year	Camel population	**Camel population density (per square kilometer)**	Human population	**Human population density (per square kilometer)**
**2011**	213320	0.11	No information available	
**2012**	213320	0.11	29,155,187	13.563
**2013**	223441	0.11	30,052,518	13.98
**2014**	210424	0.11	30,916,994	14.382
**2015**	210424	0.11	31,717,667	14.755
**2016**	481138	0.25	32,442,572	15.092
**2017**	485926	0.25	33,099,147	15.397
**2018**	490672	0.25	33,699,947	15.677
**2019**	No information available			

Note: [1] The data of camel population is from Office international des epizooties-World Organization for Animal Health Information System (OIE- WAHIS)http://www.oie.int/wahis_2/public/wahid.php/Wahidhome-/Home/indexcontent/newlang/en.

[2] The data of human population is from World Bank (WB) (a) https://data.worldbank.org.cn/indicator/SP.POP.TOTL?end=2018-&locations=SA&start=2011&view=chart; (b) https://data.worldbank.org.cn/indicator/EN.POP.DNST?end=2018&locations=SA-&start=2012.

### 2.3 Transmission model of MERS in South Korea

**Fig. 2:**
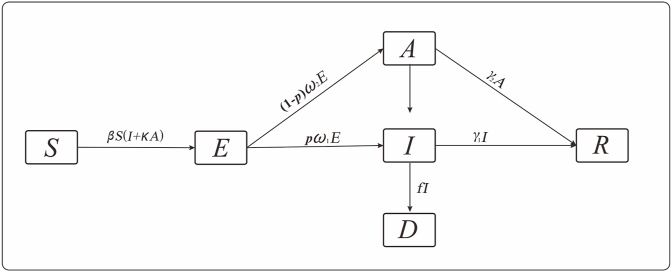
SEAIDR model based on the 2015 MERS epidemic in South Korea.

Based on the principles of the infectious disease dynamics model, we established a SEIARD model for the simulation of the transmission mode and transmissibility of MERS in South Korea. The flowchart of the model as shown in Fig. 2. The total population was divided into susceptible (*S*), exposed (*E*), symptomatic infected (*I*), asymptomatic infected (*A*), death (*D*), and recovered population (*R*) groups. The model was based on the following assumptions:

a) During the outbreak, natural birth and death rates were low compared with the size of the entire population, thus could be ignored.
b) Both symptomatic and asymptomatic infections are infectious. Thus, the susceptible could be infected following contact with both asymptomatic and symptomatic patients at transmission rates *β* and *κβ* (0 ≤ *κ* ≤ 1), respectively.
c) When susceptible individuals were infected, they became *E.*
d) The proportion of the symptomatic population was *p* (0 ≤ *p* ≤ 1), and the incubation and latent periods were 1/*ω*_1_ and 1/*ω*_2_, respectively. Therefore, after the incubation period, the *E* population could be changed to *I* or *A* at rates *pω*_1_*E* and (1*-p*)*ω*_2_*E*, respectively.
e) After the infectious period, 1/*γ*_1_, the *I* population would become *R.* However, this *I* population could also die, becoming the *D* population (case fatality rate, *f*).
f) After the infectious period, 1/*γ*_2_, the *A* population would become *R.*

The definitions of the parameters in the SEIARD model are shown in Table 3, and the equations in the SEIARD model were as follows:

$$\frac{dS}{dt}=-\beta S\left(I+\kappa A\right)$$

$$\frac{dE}{dt}=\beta S\left(I+\kappa A\right)-p\omega_{1}E-\left(1-p\right)\omega_{2}E$$

$$\frac{dI}{dt}=p\omega_{1}E-\gamma_{1}I-fI$$

$$\frac{dA}{dt}=\left(1-p\right)\omega_{2}E-\gamma_{2}A$$

$$\frac{dR}{dt}=\;\gamma_{1}I+\gamma_{2}A$$

$$\frac{dD}{dt}=fI$$

where *dS/dt*, *dE/dt*, *dI/dt*, *dA/dt, dR/dt*, and *dD/dt* represent the change rates corresponding to the groups, *S*, *E*, *I*, *A*, *R*, and *D*, respectively, at time, *t*.

**Table 3:** Parameters in SEIARD model.

Parameter	Description	Unit	Value
***β***_**1**_	Transmission rate of human-to-human	km^2^/(per*d)	–
***Κ***	Transmission rate of *A* compared with *I*	1	1
***ω***_**1**_	Coefficient of incubation period	1/d	0.1464
***ω***_**2**_	Coefficient of latent period	1/d	0.1221
***P***	Proportion of apparent infection	1	0.9301
***γ***_**1**_	Recovery rate of apparent infection	1/d	0.068
***γ***_**2**_	Recovery rate of inapparent infection	1/d	0.2
***F***	Fatality rate	1	0.1935

### 2.4 Transmission model of MERS in Saudi Arabia

**Fig. 3:**
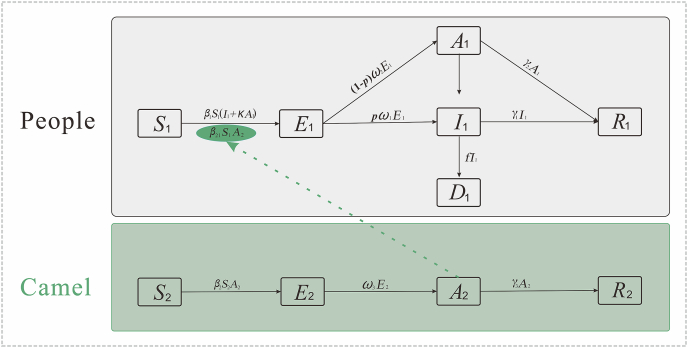
MMDM model based on the MERS epidemic in Saudi Arabia (since 2013).

Based on the principles of the infectious disease dynamics model, we established the MMDM model with an asymptomatic infection and a host animal (Fig. 3). The total human population was divided into susceptible (*S*_1_), exposed (*E*_1_), symptomatic infected (*I*_1_), asymptomatic infected (*A*_1_), recovered (*R*_1_), and death (*D*_1_) groups. Further, the total host (camel) population was divided into the susceptible (*S*_2_), exposed (*E*_2_), asymptomatic infected (*A*_2_), and recovered (*R*_2_) groups. Furthermore, the model was based on the following assumptions:

a) Person-to-person transmission was the same as for the SEIARD model in South Korea, and in addition to person-to-person transmission, people could also be infected via two other transmission routes, namely, person-to-person and camel-to-people. The transmission rates corresponding to person-to-person and camel-to-person transmission were denoted as *β*_1_ and *β*_21_, respectively.
b) The transmission rate from camel-to-camel was denoted as *β*_2_.
c) When susceptible camels were infected, they would become *E*_*2*_*.* After the latent period (1/*ω*_3_), the *E*_*2*_ camels could be changed to *A*_2_ at a rate of *ω*_3_*E*_*2*_*.*
d) After the infectious period, 1/*γ*_3_, the *A*_2_ camels would become *R*_2_.

The definitions of the parameters in MMDM model are shown in Table 4. The equations of the model are as follows:

$$\frac{dS_{1}}{dt}=-\beta_{1}S_{1}\left(I_{1}+\kappa A_{1}\right)-\beta_{21}S_{1}A_{2}$$

$$\frac{dE_{1}}{dt}=\beta_{1}S_{1}\left(I_{1}+\kappa A_{1}\right)+\beta_{21}S_{1}A_{2}-p\omega_{1}E_{1}-\left(1-p\right)\omega_{2}E_{1}$$

$$\frac{dI_{1}}{dt}=p\omega_{1}E_{1}-\gamma_{1}I_{1}-fI_{1}$$

$$\frac{dA_{1}}{dt}=\left(1-p\right)\omega_{2}E_{1}-\gamma_{2}A_{1}$$

$$\frac{dR_{1}}{dt}=\;\gamma_{1}I_{1}+\gamma_{2}A_{1}$$

$$\frac{dD_{1}}{dt}=fI_{1}$$

$$\frac{dS_{2}}{dt}=-\beta_{2}S_{2}A_{2}$$

$$\frac{dE_{2}}{dt}=\beta_{2}S_{2}A_{2}-\omega_{3}E_{2}$$

$$\frac{dA_{2}}{dt}=\omega_{3}E_{2}-\gamma_{3}A_{2}$$

$$\frac{dR_{2}}{dt}=\gamma_{3}A_{2}$$

where *dS*_1_*/dt*, *dE*_1_*/dt*, *dI*_1_*/dt*, *dA*_1_*/dt*, *dR*_1_*/dt*, *dD*_1_*/dt*, *dS*_2_*/dt*, *dE*_2_*/dt*, *dA*_2_*/dt*, and *dR*_2_*/dt* represent the change rates corresponding to the groups, *S*_1_, *E*_1_, *I*_1_, *A*_1_, *R*_1_, *D*_1_, *S*_2_, *E*_2_, *A*_2_, and *R*_2_, respectively, at time, *t*.

**Table 4:** Parameter used in S_1_E_1_I_1_A_1_R_1_D_1_-S_2_E_2_A_2_R_2_ model.

Parameter	Description	Unit	Value
***β***_**1**_	Transmission rate of human-to-human	km^2^/(per*d)	–
***β***_**21**_	Transmission rate of camel-to-human	km^2^/(per*d)	–
***β***_**2**_	Transmission rate of camel-to-human	km^2^/(per*d)	0.0142
***Κ***	Transmission rate of inapparent infection compared with apparent infection(human)	1	1
***ω***_**1**_	Coefficient of incubation period (human)	1/d	0.1923
***ω***_**2**_	Coefficient of latent period(human)	1/d	0.1429
***ω***_**3**_	Coefficient of latent period(camel)	1/d	0.1429
***P***	Proportion of apparent infection(human)	1	0.9466
***γ***_**1**_	Recovery rate of apparent infection(human)	1/d	0.0759
***γ***_**2**_	Recovery rate of inapparent infection(human)	1/d	0.2
***γ***_***3***_	Recovery rate of inapparent infection(camel)		0.0047
***F***	Fatality rate	1	0.2796

### 2.5 Parameter estimation

The parameters were estimated based on the following facts and assumptions:

a) The mean incubation and latent periods corresponding to the epidemic in South Korea were 6.83 [23,24] and 8.19 [9] days, respectively. Thus, *ω*_1_ = 0.1464 and *ω*_2_ = 0.1221. The mean incubation and latent periods for humans and the mean latent period for camels corresponding to the epidemic in Saudi Arabia were 5.2 [12,25], 7.0 [26] and 7.0 [16] days, respectively. Thus, *ω*_1_ = 0.1923, *ω*_2_ = 0.1429, and *ω*_3_ = 0.1429.
b) After examining WHO data on symptomatic MERS-CoV infections, we deduced that the proportion of such infections during the epidemics in South Korea and Saudi Arabia were 0.9301 and 0.9466, respectively.
c) The duration of disease, from illness onset to recovery, in patients with symptomatic and asymptomatic infections during the epidemic in South Korea epidemic were 14.6 [27] and 5 [8] days, respectively. Therefore, *γ*_1_ = 0.068 and *γ*_2_ = 0.2. For the epidemic in Saudi Arabia, the duration from onset of illness to recovery in the symptomatic patients was 13.17 days [28]. Owing to a lack of literature regarding the disease duration in asymptomatic patients, we set the same value in our model [8] as that corresponding to the epidemic in South Korea (5 days). Therefore, *γ*_1_ = 0.0759, *γ*_2_ = 0.2, and *γ*_3_ = 0.0047. Additionally, the data on the asymptomatic camels was fitted by the models.
d) The parameters, *β*_1_ (South Korea) and *β*_1_, *β*_2,_ and *β*_2-1_ (Saudi Arabia) were estimated by fitting the model with the collected data.

### 2.6 Quantification of the transmissibility of MERS

For each outbreak, the basic reproduction number (*R*_0_) was used to determine the transmissibility of MERS-CoV. *R*_0_ is one of the key values that is used predict whether an infectious disease will spread in a population or die out [29]. When *R*_0_ < 1, the disease will not amount to an epidemic, but will gradually disappear, with the number of infected persons decreasing monotonically to zero. Conversely, when *R*_0_ > 1, the disease will become an epidemic. For the SEIARD model, *R*_0_ was calculated according to the following equation:

$$R_{0}=\beta S\left[\frac{p}{\gamma_{1}+f}+\frac{\left(1-p\right)\kappa }{\gamma_{2}}\right]$$

For the MMDM model, *R*_0_ was calculated according the following equation:

$$R_{0}=\text{max}\left\{\frac{\beta_{2}N_{2}}{\gamma_{3}},\frac{p\omega_{1}\beta_{1}N_{1}}{\left[p\omega_{1}+\left(1-p\right)\omega_{2}\right]\left(\gamma_{1}+f\right)}+\frac{\left(1-p\right)\omega_{2}\beta_{1}\kappa N_{1}}{\left[p\omega_{1}+\left(1-p\right)\omega_{2}\right]\gamma_{2}}\right\}$$

The above equations were derived using next generation matrix methods:

$$\begin{array}{cc} F & =\left[\begin{array}{l} \beta_{1}S_{1}\left(I_{1}+\kappa A_{1}\right)+\beta_{21}S_{1}A_{2}\\ 0\\ 0\\ \beta_{2}S_{2}A_{2}\\ 0 \end{array}\right],\\ V & =\left[\begin{array}{l} p\omega_{1}E_{1}+\left(1-p\right)\omega_{2}E_{1}\\ -p\omega_{1}E_{1}+\gamma_{1}I_{1}+fI_{1}\\ \left(p-1\right)\omega_{2}E_{1}+\gamma_{2}A_{1}\\ \omega_{3}E_{2}-\omega_{3}E_{2}+\gamma_{3}A_{2} \end{array}\right] \end{array}$$

$$\begin{array}{cc} F & =\left[\begin{array}{lllll} 0 & \beta_{1}S_{1} & \beta_{1}\kappa S_{1} & 0 & \beta_{21}S_{1}\\ 0 & 0 & 0 & 0 & 0\\ 0 & 0 & 0 & 0 & 0\\ 0 & 0 & 0 & 0 & \;\beta_{2}S_{2}\\ 0 & 0 & 0 & 0 & 0 \end{array}\right],\\ V & =\left[\begin{array}{lllll} p\omega_{1}+\left(1-\rho \right)\omega_{2} & 0 & 0 & 0 & 0\\ -p\omega_{1} & \gamma_{1}+f & 0 & 0 & 0\\ \left(p-1\right)\omega_{2} & 0 & \gamma_{2} & 0 & 0\\ 0 & 0 & 0 & \omega_{3} & 0\\ 0 & 0 & 0 & -\omega_{3} & \gamma_{3} \end{array}\right] \end{array}$$

$$V^{-1}=\left[\begin{array}{lllll} \frac{1}{p\omega_{1}+\left(1-p\right)\omega_{2}} & 0 & 0 & 0 & 0\\ \frac{-p\omega_{1}}{\left[p\omega_{1}+\left(1-p\right)\omega_{2}\right]\left(\gamma_{1}+f\right)}\; & \frac{1}{\gamma_{1}+f} & 0 & 0 & 0\\ \frac{\;\left(1-p\right)\omega_{2}\;}{\left[p\omega_{1}+\left(1-p\right)\omega_{2}\right]\gamma_{2}}\; & 0 & \frac{1}{\gamma_{2}} & 0 & 0\\ 0 & 0 & 0 & \frac{1}{\omega_{3}} & 0\\ 0 & 0 & 0 & \frac{1}{\gamma_{3}} & \frac{1}{\gamma_{3}} \end{array}\right]$$

$$FV^{-1}=\left[\begin{array}{l} \frac{p\omega_{1}\beta_{1}S_{1}}{\left[p\omega_{1}+(1-\rho )\omega_{2}](\gamma_{1}+f\right)}+\frac{(1-\rho )\omega_{2}\beta_{1}\kappa S_{1}}{[p\omega_{1}+(1-\rho )\omega_{2}]\gamma_{2}}\\ 0\\ 0\\ \begin{array}{l} 0\\ 0 \end{array} \end{array}\begin{array}{l} \frac{\beta_{1}S_{1}}{\gamma_{1}+f}\\ \begin{array}{l} 0\\ 0 \end{array}\\ 0\\ 0 \end{array}\begin{array}{l} \begin{array}{l} 0\\ 0 \end{array}\\ 0\\ 0\\ 0 \end{array}\begin{array}{l} \begin{array}{l} 0\\ 0 \end{array}\\ 0\\ \frac{\beta_{2}S_{2}}{\gamma_{3}}\\ 0 \end{array}\begin{array}{l} \begin{array}{l} 0\\ 0 \end{array}\\ 0\\ \frac{\beta_{2}S_{2}}{\gamma_{3}}\\ 0 \end{array}\right]$$

$$R_{0}=\text{max}\left\{\frac{\beta_{2}N_{2}}{\gamma_{3}},\frac{p\omega_{1}\beta_{1}N_{1}}{\left[p\omega_{1}+\left(1-p\right)\omega_{2}\right]\left(\gamma_{1}+f\right)}+\frac{\left(1-p\right)\omega_{2}\beta_{1}\kappa N_{1}}{\left[p\omega_{1}+\left(1-p\right)\omega_{2}\right]\gamma_{2}}\right\}$$

### 2.7 Statistical analysis

The models were simulated and solved using Berkeley Madonna 9.1.14 (developed by Robert Macey and George Oster, University of California at Berkeley; Copyright ©1993–2001 Robert I. Macey & George F. Oster) and the fourth-order Runge-Kutta method at a tolerance level of 0.001. The goodness of fit test of the models was performed using SPSS v22.0 (IBM Corp., Armonk, NY, US) and evaluated using the coefficient of determination (*R*^2^). *P* < 0.05 was considered significant.

## 3 Results

### 3.1 Epidemiological characteristics

**Fig. 4:**
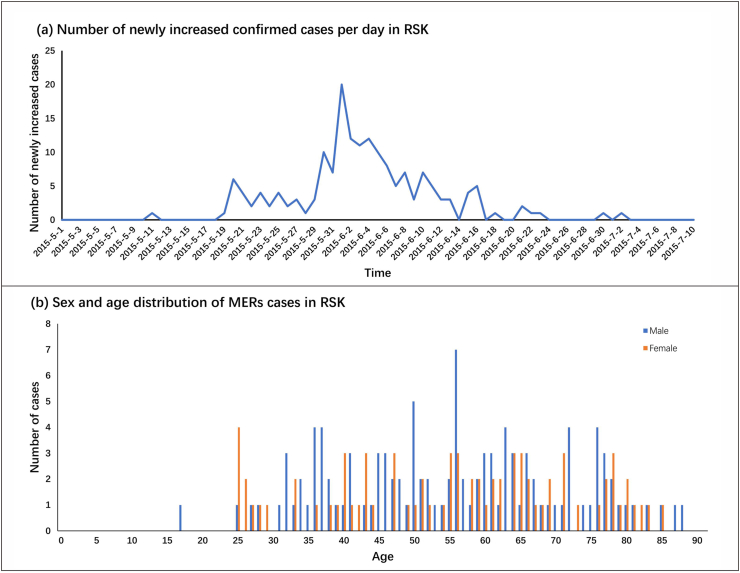
(a) Epidemic curve of newly increased confirmed case of MERS in South Korea. (b) Sex and age distribution of MERS cases in South Korea.

In South Korea, the government announced the end of the MERS epidemic on July 10, 2015, which from the date when the last case was reported (July 2, 2015), was approximately the duration of the incubation period of the disease. Overall, 187 cases, including 111 men and 86 women, with ages predominantly in the range 35–70 years, were reported (Fig. 4-(b)). No occupational data was collected, thus, it was not clear whether any of the patients was a HCW. Further, most of the cases were concentrated in Seoul and the surrounding areas, which are characterised by a high population density. It was also observed that the outbreaks were concentrated in medical institutions. On May 20, 2015, the first confirmed case of imported MERS (date of onset May 11, 2015) in South Korea was reported. Furthermore, the outbreak was concentrated within the May to July 2015 period (Fig. 4-(a)), and the number of cases peaked on June 1, 2015, after which the epidemic curve showed a gradually decreasing trend; this could possibly be attributed to the emergency response measures that were put in place by the South Korea government, such as mobilizing an emergency response team on June 8, 2015 and launching national pneumonia surveillance on June 10, 2015.

**Fig. 5:**
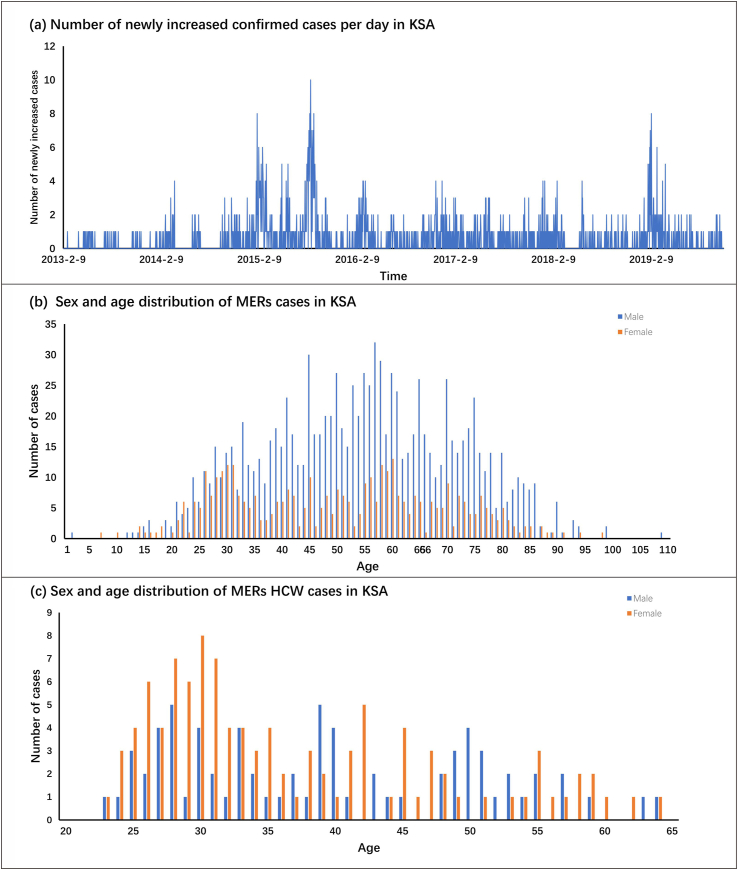
(a) Epidemic curve corresponding to newly increased confirmed MERS cases in Saudi Arabia. (b) Sex and age distribution of MERS cases in Saudi Arabia. (c) Sex and age distribution of health care workers (HCWs) MERS cases in Saudi Arabia.

For Saudi Arabia, a total 1469 MERS cases had been reported by December 18, 2019. Among these, 1455 cases with data related to age and sex distribution, included 1056 men and 399 women, whose ages were predominantly distributed within the range 35–75 years (Fig. 5-(b)) It was also observed HCW comprised 177 cases, including 72 men and 105 women, who were mainly young and middle-aged people within the 25–40 years age-group (Fig. 5-(c)). History of direct contact with camels, potential comorbidities, including diabetes and heart disease, and advanced age were identified as possible risk factors for morbidity and death during the incubation period, i.e., approximately 2 weeks before the onset of the disease. However, further research is needed in this regard. As indicated in Table 5, MERS cases have been reported for all the regions of Saudi Arabia, with the top three regions being Riyadh, Eastern, and Makkah regions (746, 183, and 145 cases, respectively). Additionally, the distribution of cases showed clustering around medical institutions and communities, and ever since the first case was reported in September 2012, cases have been reported monthly. As indicated in Fig. 5-(a), the epidemic occurs during winter and spring every year from February to April.

**Table 5:** Reported MERS cases in administrative regions of KSA.

	Province	Number of cases
**Central**	Riyadh	746
	Al-Qasim	109
**Northern**	Hail	22
	Northern Border	12
	Al-Jawf	34
**Western**	Makkah	145
	Madinah	43
	Tabuk	13
	Al-Bahah	8
**Eastern**	Eastern	183
**Southern**	Jizan	4
	Najran	70
	Assir	50

### 3.2 Model results

**Fig. 6:**
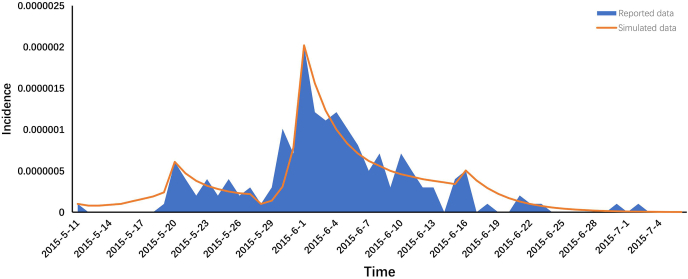
Comparison of simulated and actual MERS outbreak in the SEIADR model for the epidemic in South Korea.

According to the SEIARD model for South Korea, the model parameters, and initial values recorded in Table 3, the simulation results (Fig. 6) showed that the development of the epidemic gradually increased from May 11, 2015 and accelerated on May 28, 2015, peaking on June 1, 2015, and thereafter showing a declining trend. Further, the entire outbreak lasted approximately 55 days, which could be divided into five intervals based on the trends in the number of daily new cases. The fitted data was tested for goodness of fit with the actual outbreak data. Thus, the coefficient of determination, *R*^2^, obtained was 0.844, and the differences between the model data and the actual data were not significant (*P* > 0.05), indicating that the model fitting effect was ideal. Further, the *R*_0_ values ranged from 0 to 28.02, with a mean of 8.59 (Table 6).

**Table 6:** Parameter estimation of the SEIADR model and the results of *R*_0_.

	*b*_1_	*R*_0_	*R*_0AVE_(95%CI)
**2015.05.11**–**2015.05.20**	1.2490	4.88	8.59 (0–28.02)
**2015.05.20**–**2015.05.28**	0.2610	1.02	
**2015.05.28**–**2015.06.01**	9.3125	36.38	
**2015.06.01**–**2015.06.16**	0.1668	0.65	
**2015.06.16**–**2015.07.06**	4.5868 × 10^−8^	1.79 × 10^−7^	

**Fig. 7:**
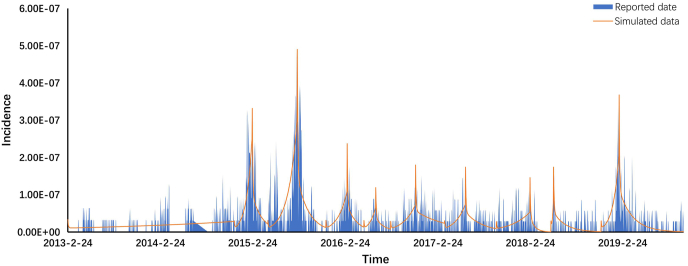
Comparison of simulated and actual MERS outbreak in the MMDM for the epidemic in Saudi Arabia.

Furthermore, based on the MMDM for Saudi Arabia, the model parameters, and the initial values provided in Table 7, the simulation results (Fig. 7) showed that from 2012, the development of the epidemic in Saudi Arabia exhibited a gradually decreasing seasonal trend. Testing the model data for goodness of fit with the actual outbreak data, the coefficient of determination, *R*^2^, was 0.507, and the differences between these two datasets were not significant (*P* > 0.05), indicating that the model fitting effect was ideal. The range of *R*_0_ values corresponding to person-to-person transmission was 0.86–1.44 (mean = 1.15), and that corresponding to camel-to-camel transmission was 1.02.

**Table 7:** Parameter estimation of the S_1_E_1_I_1_A_1_D_1_R_1_-S_2_E_2_A_2_R_2_ model and the results of *R*_0_.

	*b*_1_	Person-to-Person *R*_0_	Person-to-Person *R*_0AVE_ (95%CI)	*b*_2_	Camel-to-Camel *R*_0AVE_
**2013.02.24**–**2014.12.13**	0.3490	1.01	1.15 (0.86–1.45)	0.0142	1.02
**2014.12.13**–**2015.02.22**	0.4854	1.41			
**2015.02.22**–**2015.04.25**	0.2766	0.80			
**2015.04.25**–**2015.08.19**	0.4320	1.25			
**2015.08.19**–**2015.12.10**	0.2839	0.82			
**2015.12.10**–**2016.03.03**	0.4289	1.24			
**2016.03.03**–**2016.05.08**	0.2749	0.80			
**2016.05.08**–**2016.06.24**	0.4661	1.35			
**2016.06.24**–**2016.08.22**	0.2735	0.79			
**2016.08.22**–**2016.11.28**	0.4072	1.18			
**2016.11.28**–**2017.03.23**	0.3233	0.94			
**2017.03.23**–**2017.06.13**	0.4180	1.21			
**2017.06.13**–**2017.10.13**	0.3039	0.88			
**2017.10.13**–**2018.02.23**	0.3838	1.11			
**2018.02.23**–**2018.05.17**	0.2067	0.60			
**2018.05.17**–**2018.05.27**	1.1866	3.44			
**2018.05.27**–**2018.12.01**	0.2880	0.84			
**2018.12.01**–**2019.02.10**	0.4765	1.38			
**2019.02.10**–**2019.11.06**	0.2932	0.85

### 3.3 Analysis of the impact of the ‘host-to-human’ transmission route

As shown in Table 8, we set different scenarios for different values of β_1_ and β_21_ to simulate and compare the impact of the ‘Host-to-Human’ transmission route on all possible MERS-CoV transmission routes. The results obtained showed that when the infectivity coefficient, b_1_, in the ‘Human-to-Human’ route was assumed to be null, there were no significant changes in the morbidity of MERS regardless of whether the infectivity coefficient, b_21_, corresponding to the ‘Host-to-Human’ route was null or not. Conversely, when b_21_ was kept constant, a change in the value of b_1_ could lead to a significant change in the total attack rate (TAR). Therefore, the ‘Host-to-Human’ transmission route may be one of the potential MERS-CoV transmission routes; however, it is not the main transmission route as verified by the model simulation results.

**Table 8:** Comparison of TAR in different situations in KSA.

	b_1_≠0, b_21_≠0	b_1_ = 0, b_21_≠0	b_1_≠0, b_21_ = 0	b_1_ = 0, b_21_ = 0
**TAR**	9.84 × 10^−5^	8.43 × 10^−6^	9.84 × 10^−5^	8.43 × 10^−6^

## 4 Discussion

MERS is a kind of respiratory infectious disease that is characterised by seasonality and periodicity. However, its transmission route is complicated and unknown, and moreover, in some cases, it is asymptomatic. Reportedly, identifying the natural host of emerging human-infected pathogens is the first step in preventing their transmission and developing corresponding control measures. For MERS-CoV, unlike the imported route that characterized the epidemic in South Korea, some of the outbreaks in Saudi Arabia could be attributed to more than a single source. Further, considering that MERS-CoV is possibly an animal-derived pathogen and given that numerous studies highlight direct contact with camels as one of risk factors for human MERS-CoV infections, we innovatively considered the “Camel-to-People” transmission route as a potential transmission route and established the multi-population and multi-channel ordinary differential equation models, namely, SEIARD model and MMDM, respectively, to explore the epidemic characteristics of MERS-CoV and quantify its transmissibility (*R*_0_). Notably, theoretical epidemiological models can help overcome the over-reliance of traditional epidemic assessment methods on expert experiences, improve the weaknesses of other mathematical modelling methods, such as complexity and the need for professional judgement, and then establish a scientific method that can be used for disease control and for the evaluation of outbreaks without adequate expert resources.

Therefore, in this study, to evaluate the applicability of the model, we utilised the goodness of fit test, *R*^2^, and curve-fitting, all of which showed ideal fitting. Specifically, the fitting results corresponding to the SEIARD model showed that the transmissibility of MERS-CoV during the epidemic in South Korea was strong, with *R*_0_ = 8.59 (95% CI: 0–28.02). Comparatively, the results corresponding to the MMDM showed weak transmissibility during the epidemic in Saudi Arabia, with the *R*_0_ values corresponding to ‘Person-to-Person’ and ‘Camel-to-Camel’ being 1.15 and 1.02, respectively (95% CI: 0.86–1.44). Additionally, compared with the transmissibility of the SARS epidemic in mainland China in 2003 and the COVID-19 pandemic in early 2020, the transmissibility of MERS-CoV during the epidemic in South Korea in 2015 was higher, whereas that corresponding to the epidemic in Saudi Arabia was lower. The transmissibility of MERS-CoV corresponding to the ‘Person-to-Person’ transmission route during the epidemic in Saudi Arabia was similar to those reported in other studies.

**Fig. 8:**
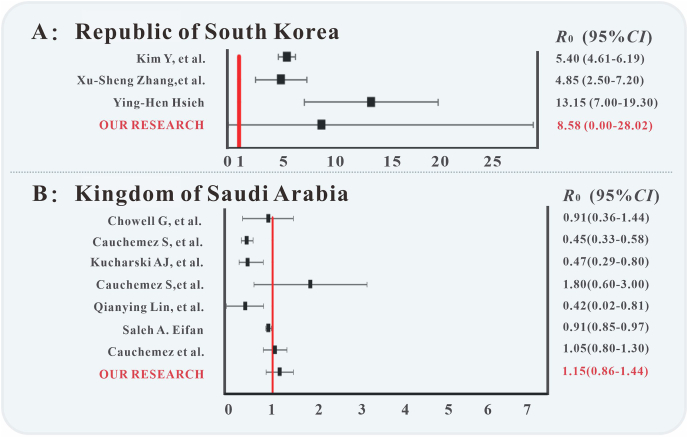
Forest plot of *R*_0_ of MERS (A, Republic of South Korea; B, Kingdom of Saudi Arabia).

We searched PubMed database (Link: https://www.ncbi.nlm.nih.gov/pubmed) for related articles, using keywords such as: “MERS”, “MERS-CoV”, or “Middle East Respiratory Syndrome” and “Model”, “Modelling”, or “Modeling”. Studies that had been cited several times and were published before June 2020 were selected. As shown in Fig. 8, the *R*_0_ of MERS-CoV obtained in this study (1.15 and 8.58 in Saudi Arabia and South Korea, respectively) was mid-range those reported in other studies, i.e., 0.5–1.0 (mean = 0.86) and 5–13 (mean 7.80) in South Arabia and South Korea, respectively [9,10,25,[29], [30], [31], [32], [33], [34], [35]], with no obvious offset or abnormal values. The transmissibility of the epidemic in South Korea considered in this study was significantly higher than those in Saudi Arabia, which is ascribed to the two main reasons as follow. Firstly, there was a spike in the number of confirmed cases around 2nd June in Fig. 4, that is, the data fluctuations in the early stage of the epidemic would have greater impacts on the model assessment of transmissibility. What's more, it happened that cluster infections and superspreading events in crowded settings such as hospitals and community in South Korea, while there are scarcely populated areas in Saudi Arabia so as to reduce the contact opportunity although the higher numbers of cases. However, it is worth noting that the higher transmissibility but low number of cases in South Korea may be attributed to the timely and effective control measures taken by the government.

Papaneri et al. [36] observed that possibly, MERS-CoV was transmitted to a camel in Africa by infected bats and then exported to the Arabian Peninsula via trade channels. Thereafter, the virus was transmitted to humans via direct or indirect contact with camels, with subsequent widespread transmission from person-to-person. With a focus on the ‘Camel-to-Human’ transmission route, the parameters, β_1_ and β_2-1_, were set in the MMDM model to simulate the process of MERS-CoV infection transmission and determine the impact of the Saudi epidemic. By establishing different scenarios for the values of β_1_ and β_2-1_ during the simulation, the result revealed that ‘Camel-to-Human’ route could be potential, but not the main MERS-CoV transmission route.

Furthermore, we constructed the theoretical epidemiological models using the basic reproduction number (*R*_0_) as a measurement indicator to quantify the transmissibility of MERS around world and explain the differences in the prevalence of MERS between regions. The before-and-after changes in the values of TAR were applied to preliminarily evaluate the effect of the “Host-to-People” transmission route.

As one of three coronavirus outbreaks that have posed serious threats to global health since the 20th century, we cannot ignore the fact that MERS cases continues to erupt under the background of the COVID-19 pandemic [38]. Additionally, research on the transmission dynamics of viruses that belong to the coronavirus family will help to provide suggestions regarding the direction of prevention and control measures, in the face of other possible new coronavirus infectious diseases in the future. First, in order to understand the epidemiological characteristics of an infectious disease epidemic, we must commence with clarifying the three distribution patterns (regional, population, and time) of the epidemic.

Specifically, regarding regional distribution, the MERS epidemic that started in 2012 predominantly occurred in the Arabian Peninsula and was concentrated in major cities and towns [39]. However, studies have shown that the transmission characteristics of MERS are affected by spatial heterogeneity [40], suggesting that the spread of MERS-CoV is also affected by other potential factors, such as population movements. In Saudi Arabia in particular, most of the movements are for religious reasons. The MERS epidemic could spread across the countries in the Saudi Arabia peninsula and even to other Muslim countries in the world, primarily owing to religious behaviour, such as pilgrimages [41]. According to incomplete statistics, millions of pilgrims cross national borders and move around the world every year [[42], [43], [44]].

Regarding the population distribution pattern of the epidemics, considering the entire population, the middle-aged and elderly population, especially the male population, accounted for the vast majority of cases. The reason for this is still unclear, but possibly, this observation could be related to the weakened immune system of the elderly [45], which is characterized by very low coordination and efficiency [46]; thus, the elderly are more vulnerable to new infections [47]. Reportedly, the average age of the patients with MERS is approximately 50 years old [48], and the mortality rate corresponding to patients aged above 80 years is close to 90%. Conversely, the mortality rate corresponding to patients aged below 20 years is only 10% [49]. In terms of gender differences in susceptibility, experiments have shown that male mice are more sensitive to SARS-CoV infection than females; however, the reason for this observation is still unclear [50]. Additionally, with respect to occupation, HCW are at higher risk of contracting MERS-CoV. Specifically, HCW accounted for 22% of all MERS infection cases, and nosocomial infections accounted for one third of all the MERS cases in Saudi Arabia [51].

Regarding time distribution, the three coronavirus epidemics all occurred in cold and dry winters, and began spreading in spring. In Saudi Arabia, the first confirmed case of MERS was reported in June 2012; however, the subsequent epidemic trend exhibited seasonality [52], with April and May showing obvious seasonal clusters [53]. Generally, it is believed that human-infected coronaviruses like SARS-CoV, MERS-CoV, and SARS-CoV-2 most likely originate from bats, which habitually live in cold and humid environments. Thus, the low temperature and low humidity that characterize winter and spring may provide favourable environmental conditions that prolong virus survival in areas where bats are concentrated [54]. However, it is worth noting that the results of some studies suggest that the coronavirus epidemic may spread at any time of the year and can last for several years [55]. However, viruses in the coronavirus family exhibit strong seasonal forcing leading to the accumulation of susceptible people in summer. This brings about an increase in the degree of transmission in the following winter, resulting in the possibility of repeated outbreaks and the possibility of a larger peak [56] during the post-pandemic period.

## 5 Limitation

Owing to limited access to data as well as other factors, this study had some limitations. First, the SEIARD model and MMDM, which were used to simulate the spread of the disease in South Korea and Saudi Arabia, respectively, from a perspective of system dynamics, are based on the assumptions that the population is homogeneous and that the contact rates and susceptibilities are consistent. Therefore, personal behaviour changes, such as during the Haji, which can cause the simulation results to deviate from the actual epidemic situation, were ignored. Second, in this study, some relevant parameters of the natural history of MERS, such as incubation period, latent period, silent-infection rate, and course of disease, were derived from existing related literature rather than from primary epidemiological survey data. Third, there is no original research data on dromedary camels, which are possible intermediate hosts of MERS-CoV, in the Middle East. Hence, sample data from existing related studies [37] were utilised. Therefore, it is possible that the natural history of the virus in camels was not realistically simulated. Lastly, the prevalence of MERS-CoV in the population was affected to a certain extent by climatic conditions, such as humidity and temperature. Further, spatial factors such as population density, were not considered in this study. Therefore, in future, it would be necessary to combine the time model with a space model for the realization of early warning on the basis of the transmission dynamics characteristics of the disease, and to provide more accurate theoretical guidance on MERS prevention and control.

## 6 Conclusion

Based on the transmission dynamics characteristics of MERS-CoV, we analysed the pattern and periodicity of the 2015 epidemic in South Korea and the 2013 epidemic in Saudi Arabia. Thus, we established the SEIARD model of ‘Person-to-Person’ transmission and the MMDM model of multi-route transmission using a sine function, respectively, to correct the seasonality and periodicity that characterize the transmission of this disease. Weekly data on disease incidence were used to calculate the infectivity coefficient of the virus β, as well as its basic reproduction number, *R*_0_. Thus, it was observed that the models could better simulate the MERS epidemic, and provide a scientific basis for a better understanding of the epidemic characteristics as well as timely early-warning. Moreover, the models showed certain reference values that can be useful for the development of targeted prevention and control measures for MERS and other emerging coronavirus epidemics around the world.

## Funding

This study was partly supported by the 10.13039/100000865Bill & Melinda Gates Foundation (INV-005834) and the 10.13039/501100001809National Natural Science Foundation (11871093).

## CRediT authorship contribution statement

**Jia Rui:** Conceptualization, Writing – original draft, Methodology. **Qiupeng Wang:** Writing – original draft, Methodology, Software. **Jinlong Lv:** Methodology, Software, Formal analysis. **Bin Zhao:** Methodology, Data curation, Visualization. **Qingqing Hu:** Writing – review & editing. **Heng Du:** Data curation, Formal analysis. **Wenfeng Gong:** Data curation, Formal analysis. **Zeyu Zhao:** Data curation. **Jingwen Xu:** Formal analysis. **Yuanzhao Zhu:** Formal analysis. **Xingchun Liu:** Data curation. **Yao Wang:** Data curation. **Meng Yang:** Visualization. **Li Luo:** Visualization. **Qiuping Chen:** Formal analysis. **Benhua Zhao:** Data curation. **Yanhua Su:** Writing – review & editing, Methodology, Data curation. **Jing-An Cui:** Writing – review & editing, Methodology, Software. **Tianmu Chen:** Conceptualization, Writing – review & editing, Methodology.

## Data Availability

All relevant data are within the paper and its Supporting Information files.
