# Supplementary material for: The transmission dynamics of Middle East Respiratory Syndrome coronavirus
Source: Travel Med Infect Dis. 2022 Jan-Feb;45:102243. doi: 10.1016/j.tmaid.2021.102243 (PMC8694792; doi:10.1016/j.tmaid.2021.102243)
Supplement: Multimedia component 1 [file mmc1.docx]

**Supplemental file：The dromedary camels in Saudi Arabia**

In March 2014, the number of MERS-CoV cases in Saudi Arabia began to increase exponentially. Compared with the South Korea epidemic in 2015, the prevalence of MERS-CoV in Saudi tend to be more complicated, except where the high population density in the several big cities like Riyadh and Jeddah be regarded as causes of aggravating the high-risk of “Person-to-Person” transmission, and the large number of camel populations living in the Middle East are also key factors that cannot be ignored (see for detail in the Table below).

**Table. The population of human and camel in Saudi Arabia, 2011-2018**

| **Year** | **The number of camels** | **Camel population density**  **（Head per square kilometer）** | **The number of humans** | **Human population density**  **（Person per square kilometer）** |
| --- | --- | --- | --- | --- |
| 2011 | 213,320 | 0.11 | No information available | |
| 2012 | 213,320 | 0.11 | 29,155,187 | 13.563 |
| 2013 | 223,441 | 0.11 | 30,052,518 | 13.98 |
| 2014 | 210,424 | 0.11 | 30,916,994 | 14.382 |
| 2015 | 210,424 | 0.11 | 31,717,667 | 14.755 |
| 2016 | 481,138 | 0.25 | 32,442,572 | 15.092 |
| 2017 | 485,926 | 0.25 | 33,099,147 | 15.397 |
| 2018 | 490,672 | 0.25 | 33,699,947 | 15.677 |

**Note：**[1] The data of camel population obtained from Office international des epizooties-World Organization for Animal Health Information System (OIE-WAHIS). Link: <http://www.oie.int/wahis_2/public/wahid.php/Wahidhome/Home/indexcontent/newlang/en>.

[2] The data of human population in Saudi Arabia obtained from World Bank. Link: (a) <https://data.worldbank.org.cn/indicator/SP.POP.TOT-L?end=2018&locations=SA&start=2011&view=chart>; (b) <https://data.worldbank.org.cn/indicator/EN.POP.DNST?end=2018&lo-cations=SA&start=2012>.
